# Interventions for obesity among schoolchildren: A systematic review and meta-analyses

**DOI:** 10.1371/journal.pone.0209746

**Published:** 2019-01-09

**Authors:** Mohamad Shariff A. Hamid, Shariff Ghazali Sazlina

**Affiliations:** 1 Unit of Sports Medicine, Faculty of Medicine, University of Malaya, Kuala Lumpur, Wilayah Persekutuan, Malaysia; 2 Department of Family Medicine, Faculty of Medicine and Health Sciences, Universiti Putra Malaysia, Serdang, Selangor, Malaysia; Weill Cornell Medical College in Qatar, QATAR

## Abstract

**Background:**

Childhood overweight and obesity has emerged as a major public health threat worldwide with challenges in its management. This review assessed the effectiveness of interventions for childhood overweight and obesity.

**Methods:**

A systematic literature search was conducted using CINAHL, EMBASE, Ovid MEDLINE, PsycINFO and SPORTDiscus databases to retrieve articles published from 1st January 2000 to 31^st^ December 2017. Randomised controlled trials (RCTs) and quasi-experimental studies comparing different strategies in managing overweight and obesity among schoolchildren (6 to 12 years of age) were included. The main outcomes of interest were reductions in weight related variables included anthropometry and body composition measurements. All variables were analysed using random effects meta-analyses.

**Results:**

Fourteen studies were reviewed, 13 were RCTs and one was a quasi-experimental study. The risk of bias for randomisation was low risk for all of RCTs except for one, which was unclear. The risk of bias for randomisation was high for the quasi-experimental study. Most interventions incorporated lifestyle changes and behavioural strategies such as coping and problem solving skills with family involvement. The meta-analyses did not show significant effects of the intervention in reducing weight related outcomes when compared with controls.

**Conclusion:**

Meta-analyses of the selected studies did not show significant effects of the interventions on weight related outcomes among overweight and obese schoolchildren when compared with controls. The role of interdisciplinary team approaches with family involvement using behaviour and lifestyle strategies to curb obesity among schoolchildren is important.

## Introduction

Childhood overweight and obesity is a serious public health problem worldwide in the 21^st^ century. The prevalence of overweight and obesity has increased in almost all countries worldwide especially in economically developed countries [1–3].

The traditional perception that a heavy child is a healthy child has changed based on evidence that overweight and obesity in childhood is associated with a wide range of serious health complications [4]. Overweight and obese children are more likely to have cardiovascular (e.g. hypertension, heart disease, high cholesterol), metabolic (e.g. type 2 diabetes), and psychosocial illnesses (e.g. eating disorders, depression and low self-esteem) than their normal-weight counterparts [1]. Also, children who were overweight and obese are at greater risk of premature illness and death in later life [5].

The United Nations International Children’s Emergency Fund (UNICEF) defined overweight and obesity as excessive and unbalanced nutrition to a point at which health is adversely affected [6]. The aetiology and pathogenesis of overweight and obesity often involve complex interaction between genetic makeup and environmental factors. Adoption of sedentary behaviour (physical inactivity, watching television and sitting in front of computer) combined with excess caloric consumption are examples of the environmental factors that are potentially modifiable in the battle against overweight and obesity [7,8].

While the fundamental principles of weight management in children might seems straightforward through reduction in energy intake and increase energy expenditure, the results of current intervention studies on childhood overweight and obesity prevention are variable. A systematic review and meta-analyses on the management of obesity among children less than 18 years of age concluded that lifestyle interventions may lead to improvements in weight and cardio-metabolic outcomes [9]. However, the evidence is limited on the optimal duration of the intervention and its long-term effectiveness. A more recent review conducted in 2015 that focused only on pre-school childhood obesity (<6 years of age) found multidisciplinary and intensive interventions have some evidence of efficacy in reducing body fat and fat mass [10]. Therefore, the objective of this review was to examine the effectiveness of interventions (including dietary, physical activity and behavioural interventions) in reducing weight related outcomes among overweight and obese children 6 to 12 years of age. It is hoped that the results from this review would provide information and guide medical practitioners and health policymakers on the management of childhood overweight and obesity.

## Materials and methods

A systematic review was conducted to explore the current approaches on managing overweight and obesity among schoolchildren. The review question was: How effective are current intervention(s) in reducing weight related outcomes including anthropometry and body composition among overweight or obese schoolchildren?” This review was registered with the International prospective register of systematic reviews (PROSPERO) CRD42016037918 [https://www.crd.york.ac.uk/prospero].

### Study selection

The study design considered in this review included randomised controlled trials (RCTs) and quasi-experimental studies. We included studies that compared strategies on the management of overweight and obesity among schoolchildren aged between 6 and 12 years of age to usual care or minimal advice. Overweight or obese were defined based on several criteria, including BMI z-scores (or standard deviation (SD) scores) [11], BMI percentile [12], BMI cut-offs based on age and gender [13] and percentage of weight for height [14]. However, studies that classified overweight or obese using other definitions were also considered. The primary outcome of this review was a change in weight related outcomes, which included anthropometry (including weight, standard body mass index (BMI) in kg/m^2^, BMI percentile, BMI z-scores and standard deviation scores, percent of overweight, weight for height percentage, waist circumference) and body composition (including lean body mass, body fat and fat mass).

The secondary outcomes measured were changes in physical activity and dietary behaviour. Physical activity assessed using physical activity questionnaires, and/or activity monitors (such as accelerometer and pedometer) as well as assessment of sedentary activities were considered in this review. Studies reporting changes in dietary intake including carbohydrate and fat intake as well as caloric estimates were included. We excluded studies that focused on interventions for preschool age groups or adolescent, interventions on prevention of obesity, on drug treatment of obesity or on normal weight children.

Studies that included school-based or non-school based (home, clinic or community) interventions were reviewed. Interventions could include one or a combination of: (1) one-to-one or group counselling or advice, (2) self-directed or prescribed physical activity programmes (with or without supervisions), (3) dietary intervention or 4) behavioural strategies. Interventions delivered by one or more providers (healthcare providers, exercise professionals, or dietitians) were considered. There was no restriction on the type and content of the control group. Interventions could be compared with usual care (no active intervention), participants listed on waiting list or those who received minimal advice.

### Data sources & search strategy

Studies were electronically searched using EBSCOhost interface for Medline, CINAHL, Psychology and Behavioural Sciences Collection and SPORTDiscus, and EMBASE databases. We adhered to the Preferred Reporting Items for Systematic reviews and Meta-Analyses (PRISMA) guidelines [15]. The search strategy performed using the Medical Subject Heading (MeSH) terms and keywords. For children, search was done using the MeSH term child and the keywords child$ OR children. For overweight we used a combination of MeSH terms as follows: overweight OR obesity OR pediatric obesity. Diet [MeSH] OR diet, food and nutrition [MeSH] were used for diet and exercise [MeSH] OR physical activity for exercise and behavior therapy [MeSH]. For anthropometry we used body mass index [MeSH] OR body composition [MeSH] OR anthropometry [MeSH].

Peer-reviewed published articles between 1st January 2000 and 31^st^ December 2017 were used. Published systematic reviews on the management of obesity among schoolchildren were used as the source of randomised controlled trials (RCTs). Potential eligible studies were hand searched from the reference lists of review articles and included studies. We limited the search to include studies that involved children between 6 and 12 years of age. All the titles, abstracts, and full-text of each study retrieved from the search were screened by both reviewers using a standardized form for study eligibility. In cases where there was any doubt on the paper eligibility, the issue was resolved through discussion until a consensus was reached. In view of limited resources for translation, articles that were published in the English language were considered in this review.

### Data extraction

The titles and abstracts of all studies retrieved were reviewed following the criteria for study selection to decide if the full-text manuscripts were required for further evaluation. Each full-text article retrieved was evaluated systematically according to the study’s: (1) objective (on the effectiveness of interventions), (2) characteristics of the study (study design, participants’ age, behavioural theoretical model, and sample size), (3) contents of the intervention (intervention strategies, intervention provider, length of intervention and follow-up contacts), (4) targeted outcome/s and (5) major findings.

### Methodological quality assessment of individual studies

Each selected study was evaluated for its methodological quality using the Cochrane Collaboration tool for assessing the risk of bias (the Cochrane Handbook for Systematic Review of Interventions) [16]. It covers: a) sequence generation b) allocation concealment c) blinding d) incomplete outcome data (e.g. dropouts and withdrawals) e) selective outcome reporting and f) other areas of bias. For each domain in the tool, the procedures undertaken for each study were described. Each study was rated as ‘high’, ‘low’ or ‘unclear’ risk of bias based on a judgement of the gathered information. These judgements were made independently by two review authors based on the predetermined criteria and later discussed in a meeting until a consensus achieved.

### Data synthesis and analyses

We conducted a narrative synthesis based on the primary and secondary outcomes of this review. The primary outcome measures were pooled and calculated using the statistical software RevMan 5.3, according to the Cochrane Handbook for Systematic Reviews of Interventions [16]. Attempts to contact the authors to obtain the raw data for data analysis was made but to no avail. The results of the BMI z-score, waist circumference and body fat percentage were analysed using weighted or standardized mean differences as a measure of effect size, with 95% confidence intervals. Since the participant demographics and clinical settings differed among studies, we assumed the presence of heterogeneity a priori. Therefore, we used a random-effects model to pool the results. We assessed heterogeneity using the Cochran’s Q statistic of heterogeneity with reported p-value and the degree of inconsistency across studies was quantified using I^2^. In studies with three arm RCTs, each of the intervention group was analysed independently and compared with the control group. A funnel plot was performed to determine the presence of potential publication bias using the statistical software RevMan 5.3.

## Results

### Literature search

**Fig 1:**
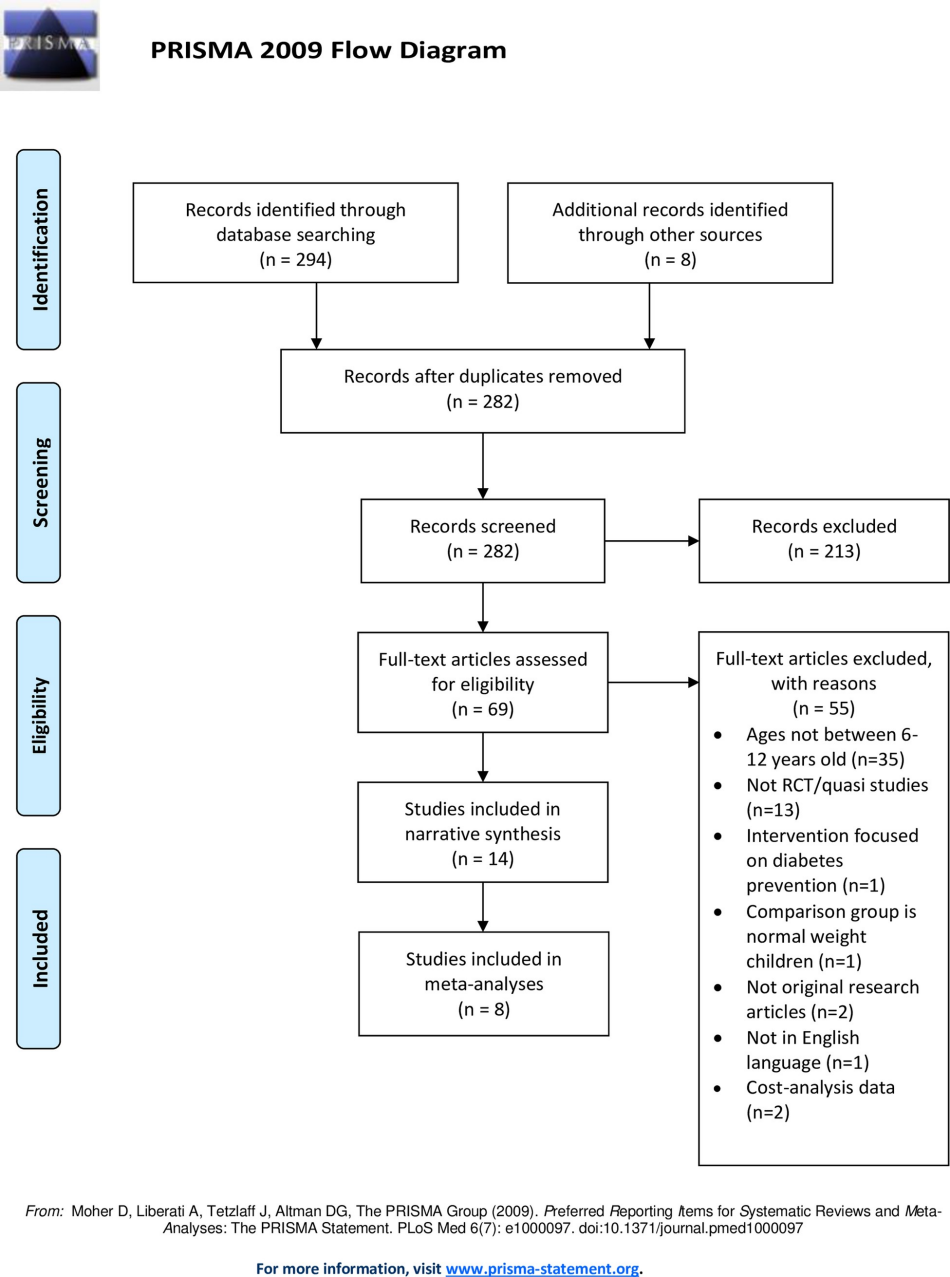
Flow diagram for selected studies.

Three hundreds and two articles were identified through the five databases and cross referencing. Twenty were removed due to duplication. After screening the titles and abstracts, 69 full-text articles were retrieved and assessed for eligibility. Fifty-five articles were excluded because they did not fulfil the selection criteria. The reasons for exclusion included participants age were not between 6 and 12 years of age (n = 35), studies identified were neither RCT nor quasi (n = 13), the comparison group was with normal weight children (n = 1), intervention focused on diabetes prevention (n = 1), not an original research article (n = 2), not in English language (n = 1), and data presented was on cost-analysis (n = 1). A total of 14 articles were included in the narrative synthesis and eight were included in the meta-analyses. The flow diagram for the study selection is described in Fig 1.

### Characteristics of selected studies

Table 1 summarises the characteristics of the selected studies. Thirteen RCTs [17–29] and one three-arm quasi-experimental study were reviewed [30]. Three RCTs were conducted in North America [17,22,25], three in Israel [27,28,30], the remaining studies were conducted in Netherlands [18], Norway [25], Finland [21], New Zealand [23], United Kingdom [29], Australia [20] and Malaysia [24]. As for the study setting, three studies were conducted in the community [27–29], five in the hospitals [18–20,26,30], and three in the clinics [17,24,25], of which two were in academic research clinics [17,24]. Two studies were home-based [22,23], and another was conducted in school [21].

**Table 1:** Characteristics of selected studies.

Author, year, country	Participants, Sample size	Intervention/ Control	Intervention length, Follow-up (FU) from baseline, Retention rate	Intervention provider	Outcomes	Significance difference between groups
**RCT, Hospital-based**						
Kalarchian et. al., 2007, USA	• Age: 8–12 years • Obese (BMI percentile ≥97^th^), no comorbidities & not on medications to affect weight Parent participation Sample size: 192 (IG: 97; CG: 95)	Family based intervention • 20 weekly 60-minutes group sessions for 6 months • 6 booster sessions (3 group sessions + 3 telephone calls) between 6 & 12 months • Behavioural strategies: goal setting, problem solving & self-regulation skills • PA: education to ↑ PA & ↓ sedentary activities/screen time < 15hours/week • Diet: Traffic light Eating Plan Control: usual care– 2 nutrition consultation to develop individual nutrition plan (Traffic light Eating Plan)	12 months FU: 18 months Retention: At 12 months: IG: 75.3% CG: 69.5% At 18 months: IG: 83.5% CG: 85.3%	Lifestyle coach, dietitian	Primary: 1. Percent overweight (%OW) Secondary: 1. Waist circumference 2. Body fat (DEXA scan) Did not measure PA & dietary intake Measured metabolic health outcomes: BP	Yes, for waist circumference and body fat percentage at 12 months (no measurements done at 18 months) No, for %OW at 12 and 18 months
Golley et. el., 2007, Australia	• Age: 6–9 years • Obese (BMI z-≤3.5), no comorbidities & not on medications to affect weight Parent participation Sample size: 111 (3-arm RCT) (IGP: 37; IGP+DA: 38; CG: 36)	IGP: Intensive lifestyle education • Based on Positive Parenting Program (Triple P) to promote child’s behavior on dietary + activity • 4 weekly 2-hour group sessions • 15–20 minutes of 4 weekly then 3 monthly individual telephone sessions IGP+DA: • Parent: Triple P + 7 intensive lifestyle support group sessions (family focused healthy eating) • Child: structured supervised aerobic activities (details not stated) Control: wait-listed; received general “Healthy Lifestyle” pamphlet + 3–4 5-minutes telephone call (retention strategy)	6 months FU: 12 months Retention: At 12 months: IGP: 78.4% IGP+DA: 81.6% CG: 86.1%	Dietitian, PA experts	Primary: 1. BMI z-scores Secondary: 1. Waist circumference Did not measure PA & dietary intake Measured metabolic health outcomes: BP, glucose, Lipids profile No measurements for CG at 6 months for comparison	No, for BMI z-score & waist circumference at 12 months
**RCT, Hospital-based**						
di Niet et. al., 2012, Netherlands	• Age: 7–12 years • Overweight (BMI SDS >1.1) or obese (BMI SDS >2.3) • no comorbidities Parent participation Sample size: 114 (IG: 73; CG: 68)	Self-monitoring of lifestyle behaviour via a short message service maintenance treatment (SMSMT) using mobile phones with personalized feedback • 8 group sessions in 3 months • Behavioural strategies: coping skills, goal setting, problem solving & self-regulation skills • PA: exercise behavior–content was not stated • Diet: healthy eating–content was not stated Control: Same content as IG but no SMSMT	3 months FU: 12 months Retention: Measured at 12 months only: IG: 86.3% CG: 69.1%	Psychologist, dietitian, paediatrician & physiotherapist	Primary: 1. BMI SDS (z-scores) Secondary: 1. Eating behavior (Dutch Eating behavior questionnaire) Did not measure PA	None for BMI SD scores or eating behavior
Hystad et. al. 2013, Norway	• Age: 7–12 years • Obese (BMI z-scores ≥2) • No comorbidities Parent participation Sample size: 99 (IG: 47; CG: 52)	Therapist-led group (TLG) to enhance parental competence to accomplish targeted lifestyle changes • 10 group sessions + 5 individual sessions on diet and PA • Written manual • Physical activity: at least 1 hour/day of moderate intensity & max. 2 hour/day sedentary behavior • Diet: Healthy eating Control: Self-help group (principle of mutual help, derived from the participants’ own experiences and knowledge. No education or guidance to improve weight	6 months FU: 24 months Retention: At 6 months: IG: 89.3% CG: 90.4% At 24 months: IG: 76.6% CG: 84.6%	Psychologists, dietitians, paediatricians & physiotherapists	Primary: 1. Body fat (DEXA scan) 2. BMI z-scores 3. Dietary intake (4-day food record: Norwegian Food composition table) Did not measure PA	None for body fat, BMI z-score and diet
**RCT, Clinic-based**						
Estabrooks et. al., 2009, USA	• Age: 8–12 years • Overweight or obese (BMI percentile ≥85^th^) • Did not state about comorbidities Parent participation Sample size: 220 (3-arm RCT) (IGFC: 50; IGGroup: 85; IGIVR:85)	Family connection (FC) Interventions • FC-workbook: self-help connection 61-pages workbook for parents & served as control group • FC group: workbook + 2-hours x 2 weekly group sessions with dietitian • FC interactive voice response (IVR) counselling: workbook+ group sessions +10 automated IVR tailored counselling sessions over 20 weeks • Behavioural strategies: goal setting, problem solving & role modelling • PA: education to ↑ PA & ↓ sedentary activities/TV viewing • Diet: education on healthy eating	6 months FU:12 months Retention: At 6 months IGFC:76% IGGroup:75% IGIVR: 80% At 12 months: IGFC:72% IGGroup:66% IGIVR: 74%	Dietitian (for IGGroup & IGIVR) & research team	Primary: 1. BMI z-score Secondary: 1. Dietary intake (Block Kids Questionnaire) 2. PA (Youth Behavior Risk Survey)	Yes, for BMI z-score Reduced in IGIVR compared to IG FC or IGGroup at 6 & 12 months No, for PA and dietary intake over time
**RCT, Research clinic-based**						
Wafa et. al., 2011, Malaysia	• Age: 7–11 years • Obese (BMI percentile >95^th^), no comorbidities Parent participation Sample size: 107 (IG: 52; CG: 55)	Adapted program from Scottish Childhood Obesity Treatment Trial • Parent: 8 8-hour group sessions over 26 weeks with dietitian + 1 session with clinical psychologist for behavioural strategies (self-monitoring, goal setting, problem solving & relapse prevention) • PA education (↑ PA & ↓ sedentary activities/TV viewing) • Diet: education on Traffic light eating plans) • Child: PA sessions (details not provided) Control: delayed treatment after 6 months	6 months FU: none Retention: IG: 65% CG: 84%	Dietitian, clinical psychologist. Exercise instructor	Primary: 1. BMI z-score 2. BMI Secondary: 1. PA (accelerometer) Dietary intake not measured	No, for BMI z-score & PA
Boutelle et. al., 2013, USA	• Age: 8–12 years • Overweight or obese (BMI percentile 85^th^ to 98^th^), no comorbidities Parent participation Sample size: 50 (IG: 25; CG: 25)	Guided self-help pediatric obesity (GSH-PO) • Behavioural program with12 individual sessions over 5 months (alternate week visits for 20 minutes each), • Written manual for child & parent and activities manual & self-monitoring booklets • Behavioural strategies (stimulus control, motivation, cognitive skils, social support & relapse prevention) • PA education (↑ PA & ↓ sedentary activities) • Diet: education on Traffic light eating plans) Control: delayed treatment after 5 months	5 months FU: 11 months Retention: IG: 92% at 5 months; no loss to FU at 11 months CG: 100%	Graduate student in clinical psychology	Primary: 1. BMI 2. BMI z-score 3. % OW Secondary: 1. Dietary intake (3 days 24-hour diet recall) 2. PA (accelerometer)	Yes, for BMI, BMI z-score & %OW at 5 months Yes, for BMI z-score & %OW at 11 months for IG but no control group No for PA and dietary intake over time
**RCT, School based**						
Kalavainen et. al., 2007, Finland	• Age: 7–9 years • Obese (weight for height of 120 to 200%) • No comorbidities Parent participation Sample size: 70 (IG: 35; CG: 35)	Family-centred group program focus on behavioural & solution-oriented therapy • 15 group sessions of 90 minutes • Written manual • Behavioural strategies: cognitive behavioural therapy workbook (Magnificent Kids) • PA: education on ↑ PA & ↓ sedentary activities • Diet: education on healthy diet & meal pattern Control: Routine counseling (booklet on weight management, eating habits & PA + 2 individual sessions with school nurse)	6 months FU: 12 months Retention: IG & CG: 97.1% at 6 months, no loss to FU at 12 months	Dietitian, school nurses	Primary: 1. Weight for height Secondary: 1. BMI 2. BMI SD scores (z-scores) PA or dietary intake were not measured as an outcome	Yes, for weight for height, BMI & BMI-SDS at 6 months Yes, for weight for height & BMI at 12 months
**RCT, Home-based**						
Goldfield et al., 2006, Canada	• Age: 8–12 years • Overweight (BMI percentile 85^th^ to 94^th^) or obese (BMI percentile >94^th^) • Watching TV (VCR/DVD or video games) >15 hours • <30 minutes physical activities • No condition that limits PA Parent participation Sample size: 30 (IG: 14; CG: 16)	Open-loop feedback (requires person to do PA, accelerometer measure PA objectively, which provide feedback. Child will be rewarded access to television when perform PA) + reinforcement by a parent • Bi weekly meeting with research team to determine the amount of television time based on the accelerometer • PA accumulated will be rewarded with access to television • Diet: None Control: Open-loop feedback only	8 weeks FU: none Retention: 100% both groups	Not stated	Primary: 1. BMI Secondary: 1. PA (accelerometer) 2. PA (Past Day PA Recall + television viewing time) 3. Dietary intake (3 days 24-hour diet recall)	Yes, for BMI, PA, television viewing time, fat intake, calories from snacks &snack intake during television watching
Maddison et al., 2014, New Zealand	• Age: 9–12 years • Used electronic media (≥15 hours per week,) • Overweight or obese (as per Cole International cut-points) • No condition that limits PA Parent/primary caregiver participation Sample size: 251 (IG: 127; CG:124)	Screen-Time Weight-loss Intervention Targeting Children at Home(SWITCH) • One face-to-face individual education and support for primary caregivers to reduce media use at home. • Monthly e-newsletter on reduced screen-based activity & links to community-based programs • Behavioural strategies: self-monitoring, role modelling • PA: focus on sedentary behavior • Diet: none Control: usual care	24 weeks FU: None Retention: IG: 95% CG: 94%	Primary caregivers	Primary: 1. BMI z-scores Secondary: 1. BMI 2. Waist circumference 3. Body fat (BIA) 4. PA (7-day physical activity questionnaire + sedentary activity) 5. Dietary intake (Food frequency questionnaire)	None for BMI z-scores, BMI, waist circumference, body fat, PA (including sedentary time) and dietary intake
**RCT, Community-based**						
Nemet et al., 2008, Israel	• Age: 8–11 years • Obese (BMI percentile >95^th^) • No comorbidities • Not on medications that interfere with growth or weight control Parent participation Sample size: 22 (IG: 11; CG:11)	• PA: 2x a week of 1-hour training group session + 1x/week movement therapy group session at Sport Centre + 30–45 minutes home-based walking/weight bearing exercise 2x/week + reduce sedentary behaviour • Diet: 14 sessions with dietitian on food pyramid with written dietary information & received balanced hypocaloric diet Control: 1 x nutritional consultation & instructed to perform physical activity three times per week on their own	3 months FU: None Retention: 100% for both groups	Dietitian, sports trainers, movement therapists	Did not specify which outcomes as primary or secondary outcomes 1. BMI percentile 2. BMI 3. Body fat (BIA) 4. Screen time 5. Diet (2 day 48-hour diet recall)	Yes, for BMI percentiles & screen time
Nemet et al., 2013, Israel	• Age: 7–9 years • Obese • (BMI percentile >98^th^) • No comorbidities • Not on medication causing obesity Parent participation Sample size: 45 (IG: 25; CG: 20)	• PA: 2x a week of 1-hour training group session + 1x/week movement therapy group session at Sport Centre + 30–45 minutes home-based walking/weight bearing exercise 2x/week + reduce sedentary behavior • Diet: 14 sessions with dietitian on food pyramid with written dietary information & received balanced hypocaloric diet Control: one nutritional consultation and instructed to perform physical activity daily	3 months FU: None Retention: IG: 88% CG: 90%	Dietitian, sports trainers, movement therapists	Did not specify the primary or secondary outcomes 1. Weight 2. BMI percentile 3. BMI 4. Skinfolds (triceps & subscapular) 5. Body fat (BIA) 6. Physical activity pattern (METS) 7. Screen time	Yes, for weight, BMI, BMI percentile, skinfolds & PA
Sacher et al., 2010, UK	• Age: 8–12 years • Obese (BMI percentile ≥ 98^th^) • No comorbidities Parent participation Sample size: 116 (IG: 60; CG:56)	Mind, Exercise, Nutrition, Do it (MEND) program • Group based sessions for 9 weeks at community venues • 12-weeks of free family swim pass • Behavioural strategies: 8 sessions in 9 weeks on stimulus control, goal setting, reinforcement, response prevention • PA: 18 group exercise sessions for 1 hour, over 9 weeks • Diet: 8 group nutrition education sessions on healthy eating with written instructions Control: Wait-list	6 months FU: 12 months Retention: IG: 61.7% CG: 80.3%	Dietitians, Health trainers	Did not specify the primary or secondary outcomes 1. Weight 2. Waist circumference 3. Lean body mass (BIA) 4. Fat mass (BIA) 5. Body fat (BIA) 6. BMI z-score 7. BMI 8. Physical activity (hour/week) (non-validated questionnaire + sedentary activity) Measured metabolic health outcomes: BP	Yes, for waist circumference, BMI z-score, BMI & sedentary activity at 6 months Yes, for waist circumference, BMI z-scores & PA at 12 months but no comparison control group
**Quasi-experimental, Hospital-based**						
Shalitin et. al. 2009, Israel	• Age: 6–11 years • Obese (BMI percentile >95^th^) • No comorbidities • Not using medication that might interfere with weight control Sample size: 162 (IGEx = 52, IGDiet = 55, IGDietEx = 55)	IGEx: Exercise intervention (3-day weekly, 90 minutes per training group session) directed by 3 professional coaches IGDiet: Diet education with 12 weekly of 60 minutes group sessions with a dietician with written information on food pyramid and healthy eating. Also prescribed on balanced hypocaloric diet IGDietEx: Combination of both interventions	12 weeks FU: 52 weeks Retention: IGEx: 42.3% IGDiet: 49.1% IGDietEx: 50.9%	Exercise professional coaches, dietitians	Did not specify the primary or secondary outcomes 1. Waist circumference 2. Body fat (BIA) 3. BMI SDS (z scores) 4. BMI PA and dietary intake were not measured as an outcome	Yes, for BMI SDS (GDiet and IGDietEx compared to IGEx at 12 and 52 weeks) None between IGDiet and IGDietEx over time

NOTE: RCT = randomised controlled trial, BMI = body mass index, SDS = Standard deviation scores, IG = intervention group, CG = control group, PA = physical activity, SD = standard deviation, BP = blood pressure, DEXA = Dual-Energy X-Ray Absorptiometry, BIA = Bio-impedance analysis

Five studies recruited children who were overweight or obese [17,18,22,23,25], while nine recruited only obese children [19–21,24,26–30]. All studies except one excluded participants with comorbidities [25]. Most studies used BMI percentile to classify overweight or obese [17,19,22,24,25,27–30]. Three studies used BMI z-scores [18,20,26], one used weight for height percentage [21] and one used the International Obesity Task Force cut points [23] to classify overweight or obese. Interestingly, the definition used to classify overweight and obese differs in the included studies despite using the same measures. For example, some studies defined obese as BMI percentile >94^th^ centile [22,28,30] while others as BMI percentile >98^th^ centile [27,29].

All studies had interventions that integrated interdisciplinary approaches involving parent or family. Selected studies incorporated either a combination of behavioural strategies, physical activity, and dietary component [17–19,21,24,25,29], behavioural strategies and physical activity [22,23] or physical activity and dietary component without behavioural strategies [20,26–28,30]. All interventions were delivered by a group of healthcare providers (psychologist, dietitian/nutritionist, physical/sport therapist) except in one study where the intervention was delivered by a clinical psychology graduate student [17]. One study used short message services [18], while another used an automated interactive voice response system [25] to maintain behavioural change. Only five studies incorporated health behavioural theories in their interventions, namely social learning and cognitive theory [29] alone or combined with behavioural theories or models [18,23], social ecological theory [25] or the trans-theoretical model [30]. The duration of intervention varied between 8 weeks and 12 months. In nine studies participants and their families were followed up for 12 to 24 months [17–21,25,26,29,30]. Six RCTs were compared with usual care or wait-listed groups [17,19,20,23,24,29] while the others were compared with groups that received minimal advice.

All studies assessed weight related measurements as their primary outcomes. Seven studies used BMI z-scores [17,18,20,23–26] and two studies included standard BMI (kg/m^2^) [17,22] as their primary outcomes. Other studies also included body fat [26], percentage of overweight [17,19] and weight for height percentage [21] as their primary outcomes. Six studies measured body composition such as body fat, fat mass and lean body mass as their outcomes [19,23,26–29]. Five studies measured waist circumference as their outcome [19,20,23,29,30]. One study measured skinfold thickness as their outcome [27]. However, 11 studies presented more than one weight related outcomes as their outcome measures [17,19–21,23,24,26–30].

Eight studies measured changes in physical activity and sedentary behaviour including screen time [17,22–25,27–29]. In three studies, the level of physical activity was measured using the accelerometers [17,22,24]. Seven studies measured changes in dietary intake including consumptions of unhealthy snacks as outcomes [17,18,22,23,25,26,28].

### Study quality

**Fig 2:**
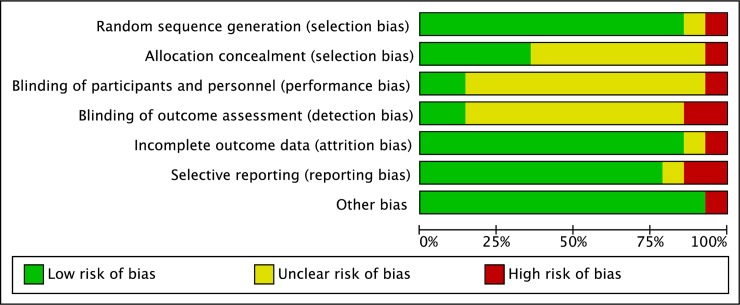
Quality assessment of selected studies.

The risk of bias for randomisation was unclear and low for all 13 RCTs [17–29]. The risk of bias for randomisation among quasi-experimental studies was high as expected. The quasi-experimental study stated random assignment of participants in their interventions. However, details on the randomisation technique was not elaborated [30].The allocation concealment was described in six (43%) studies [18,20–24]. Blinding of participant and personnel was lacking in all studies as anticipated because of the nature of the interventions. In addressing detection bias, blinding of outcome assessment was unclear in most studies. In three studies the outcome measures were performed by assessors blinded to participants’ grouping [20,22,24]. The risk for incomplete outcome data was low for all studies except for one [29]. Sacher et. al (2010) declared loss to follow-up among their participants but did not apply intention to treat analysis [29]. The proportion of studies with low, unclear and high risk of bias is presented in Fig 2.

### Effect of interventions compared with controls

Five studies that incorporated behavioural strategies with exercise and dietary interventions reported positive effects on the weight related outcomes [17,19,21,25,29]. Other studies that included behavioural skills and exercise alone [22] or combination of exercise and dietary interventions [27,28,30] also reported positive effects on the weight related outcomes. Of the seven RCTs that measured BMI z-score, four showed significant reductions among participants in the intervention compared to controls [17,21,25,29]. Three of these studies showed reductions at 6-months follow up [17,25,29], of which two studies delivered their interventions in groups sessions [25,29]. Two studies that incorporated family group sessions of behavioural strategies with exercise and dietary interventions showed no reductions in the BMI z-scores when compared to control groups [18,24]. One of these RCTs used SMS to encourage self-monitoring and provided personalised feedback [18]. Two RCTs that measured BMI percentile also showed reductions at post-intervention when compared to their controls [27,28]. Both studies incorporated group-based diet and physical activity programs with family engagement. The quasi experimental study found dietary intervention alone or combined with physical activities leads to significant reductions in BMI z-scores compared to exercise intervention alone [30].

Four out of eight RCTs showed increased physical activity in the intervention group compared to the control [22,27–29]. One study also showed reduction in sedentary activity among participants in the intervention group. The change was sustained at 6-months follow-up [29]. Seven RCTs measured dietary intake behaviour as their outcomes, which included carbohydrate and fat intake as well as estimation of caloric intake [13–16,18,19]. Only two studies showed significant reductions in dietary or caloric intake when compared to the control groups at post-intervention, however, they did not follow up the participants [22,23]. One study showed reductions in fat and caloric intake from snacks and reduction in snack intake during watching television [22].

All community-based intervention studies reported positive effects on changes of BMI z-score, BMI percentile, waist circumference and/or skinfolds, as well as improvement in sedentary behaviours [27–29]. However, only one study followed participants up to 12 months and showed sustained effects on these outcomes [29]. One of the three hospital-based studies [29], one of the two home-based [22], and each of the clinic-based [17] and school-based [21] interventions reported positive effects on the outcomes.

**Fig 3:**
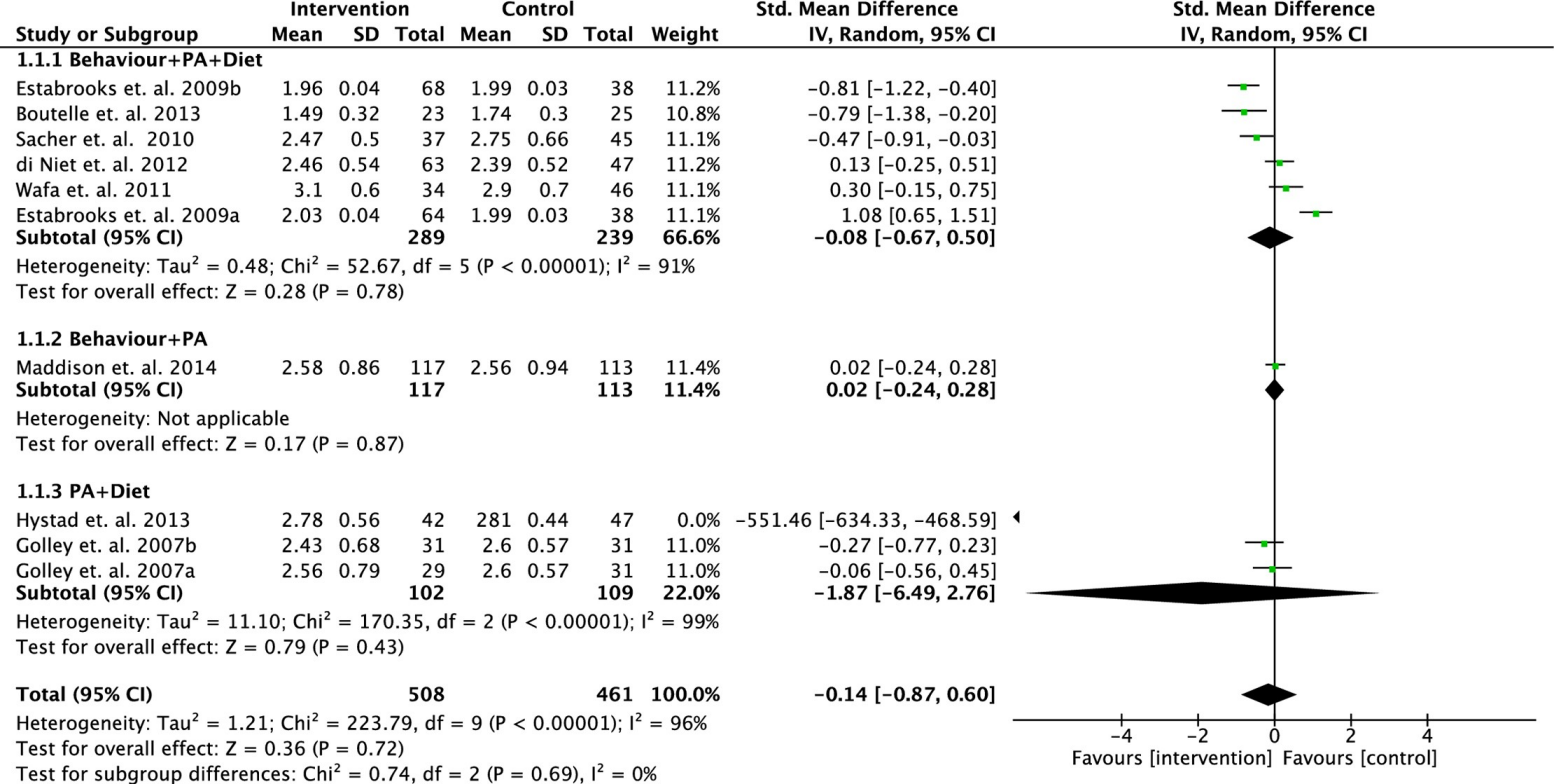
Forest plot on BMI z-score based on intervention strategies at postintervention.

**Fig 4:**
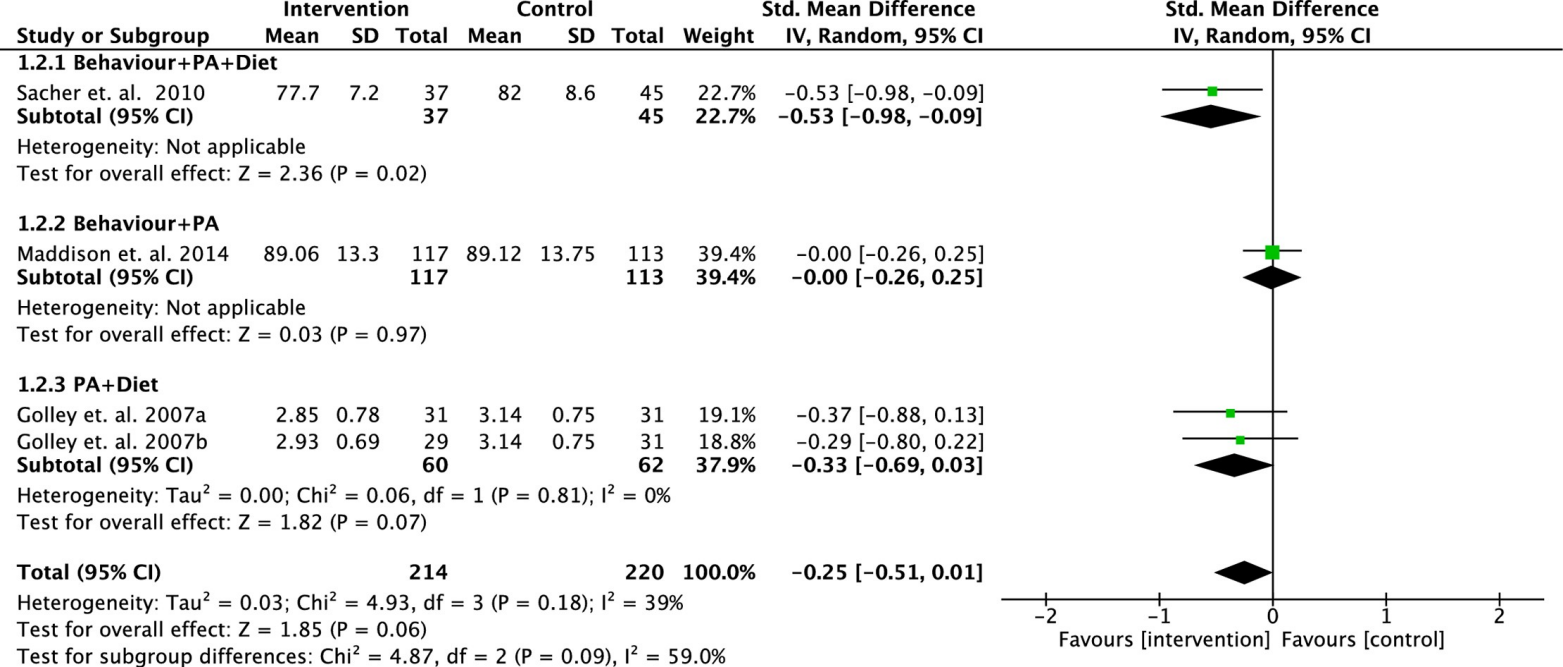
Forest plot on BMI percentile based on intervention strategies at postintervention.

**Fig 5:**
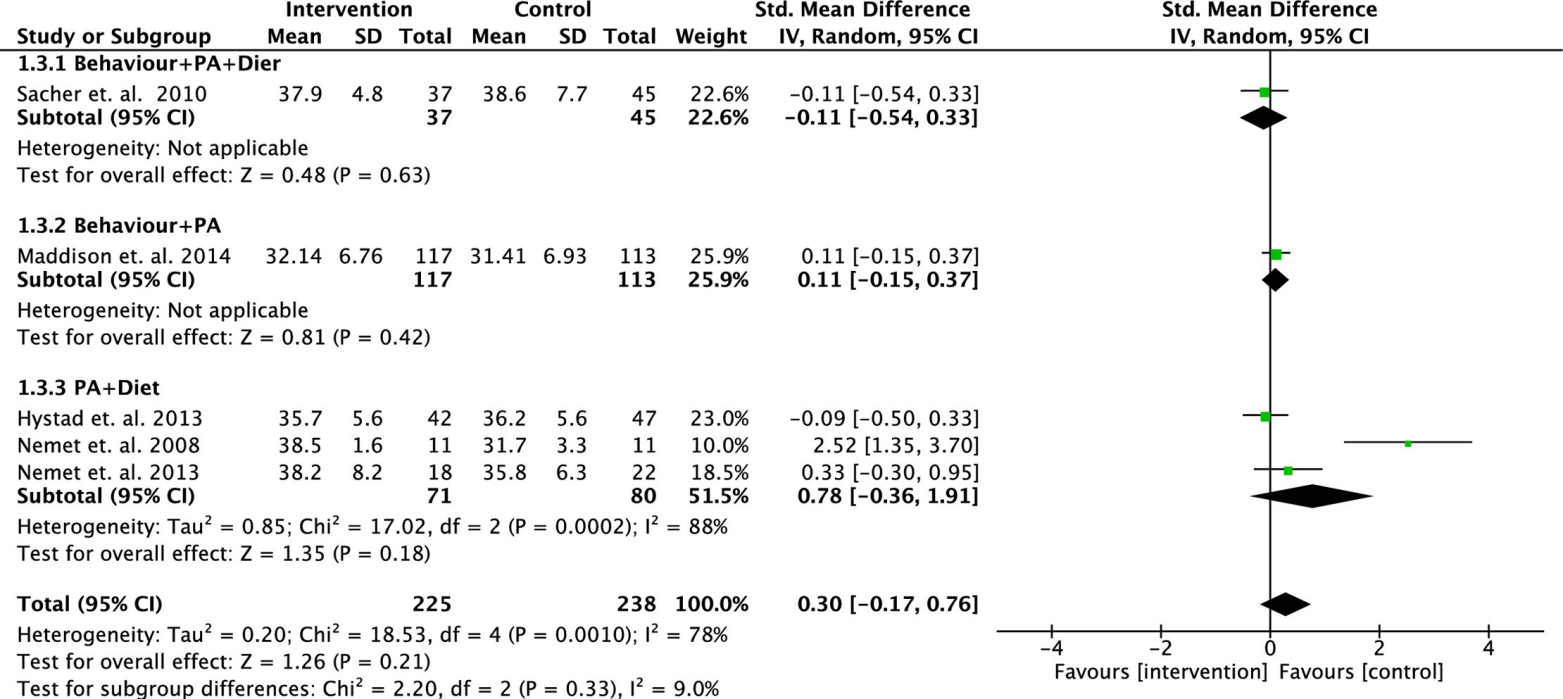
Forest plot on body fat percentage based on intervention strategies at postintervention.

We performed meta-analyses to determine the effect of interventions on changes in BMI z-score, waist circumference and body fat percentage based on different intervention strategies. Some of the selected studies varied in their primary outcomes, therefore, data from studies with similar outcomes were pooled and analysed. Data from eight RCTs (n = 969 participants) were pooled to determine the effects of intervention strategies on the changes of BMI z-scores. However, two of the studies were 3-arm RCTs, which we analysed each intervention separately. No statistically significant difference was found between intervention and controls (standardised mean difference = - 0.14; 95% CI = - 0.87, 0.60; p = 0.72) (see Fig 3) [17,18,20,23–26,29]. A meta-analyses on three RCTs (n = 434 participants) did not show significant positive effects of any intervention strategies on the changes of waist circumference compared to controls (standardised mean difference = -0.25; 95% CI = -0.51, 0.01; p = 0.06) (see Fig 4) [20,23,29]. Five RCTs (n = 463 participants) pooled data showed no significant difference in the changes of body fat percentage between intervention and control (standardised mean difference = 0.30; 95% CI = - 0.17, 0.76; p = 0.21) (see Fig 5) [23,26–29].

**Fig 6:**
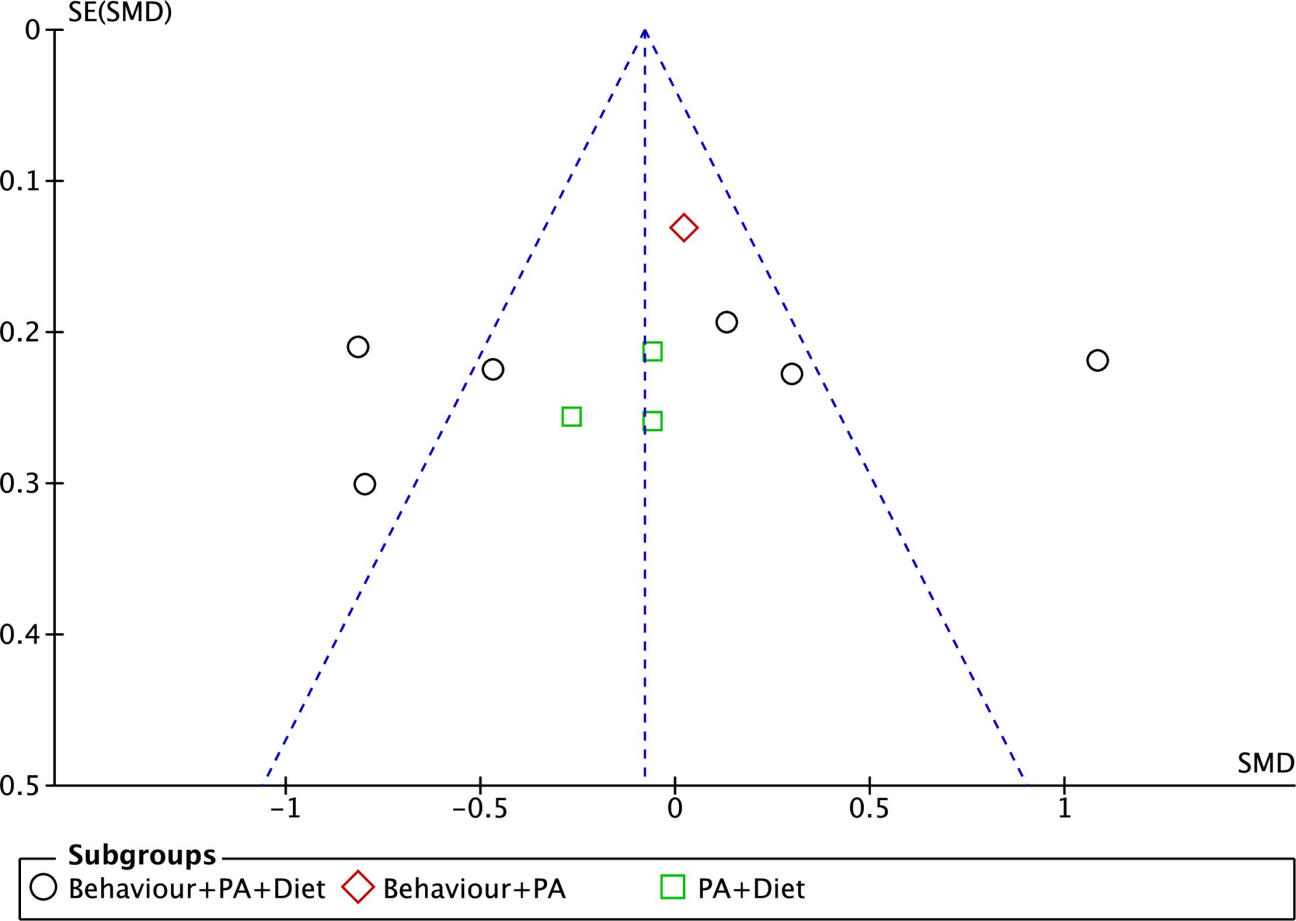
Funnel plot on BMI z-score based on intervention strategies at postintervention.

We only present a funnel plot for the BMI z-score outcome but not for the BMI percentile and body fat percentage. This is because a funnel plot with fewer than 10 studies in a meta-analysis would lead to low power of analysis to distinguish the chance from real asymmetry [31]. Fig 6 depicts the funnel plot for the meta-analysis on the effects of the intervention on the changes in BMI z-score of the selected studies in our review. There was an asymmetry of the plot to suggest presence of publication bias.

## Discussion

We conducted this review to evaluate the effectiveness of interventions on weight related outcomes (BMI z-score, BMI percentile and body fat percentage) and lifestyle outcomes (physical and sedentary activities, and dietary behaviour) among overweight or obese schoolchildren.

Our narrative synthesis found studies that incorporated behavioural lifestyle interventions reported positive effects on weight related outcomes. Previous reviews and meta-analyses reported interventions that incorporate lifestyle modifications including dietary restriction, physical activities (exercise) alone or in combination leads to larger effects on BMI, BMI z-scores and body composition, as well as reduced unhealthy dietary intake when compared to controls [9,32,33]. Moreover, a previous systematic review found addition of pharmacological intervention to behavioural lifestyle intervention led to only small effects on BMI and BMI z-score. [33]. Hence, this emphasised that behavioural lifestyle modification is utmost important in the management of obesity in children.

Studies that integrated behavioural skills into the lifestyle modifications intervention showed positive effects on the reductions of BMI z-scores, BMI percentile, waist circumference and body fat, as well as improved physical, sedentary and dietary behaviours. The parents of children in the trials were taught coping and problem-solving skills, which could have facilitated the weight reduction, hence, improved outcomes. A previous case control study that examined family functioning, expressed emotion and coping skills found mothers’ negative expressed emotion and coping skills were related to the child being overweight [34]. Hence, such behavioural skills should be incorporated as strategies in the management of obesity in children.

In this review, all studies that engaged family members in their intervention showed positive effects on weight related outcomes. Such findings are consistent with previous reviews which reported family based interventions with parental involvement led to reductions in BMI, BMI z-scores and body composition [32,35]. The engagement of parents in these trials facilitated their children in choosing healthier behaviour in addition to acting as role models for their children.

In most studies with positive effects, the delivery of the intervention involved interdisciplinary approaches involving various healthcare practitioners including dietitians, psychologists and physical or sports therapists. Moreover, in some of these studies, intervention sessions were conducted in groups. A previous systematic review on childhood obesity showed that behavioural lifestyle interventions delivered by trained specialised interventionists were effective for obesity [36]. Continuous external support from other people, or professionals may be important in achieving and maintaining goals.

Our review found that the studies were set in various settings, including in the community, school, home and the hospital. All community-based interventions reported positive effects on the primary outcomes. Our findings are consistent with previous systematic reviews which reported positive outcomes from combined lifestyle interventions (diet and physical activity) delivered in the community [36]. It was anticipated that community-based interventions would be more cost-effective compared to clinic or hospital settings. An economic evaluation on 10 RCTs reported lifestyle interventions are potentially cost-effective for obese children between 10 and 11 years of age. Its impact on health benefits and cost-savings however, would only be evident in their 6^th^ or 7^th^ decade of life [37].

The outcome measured in majority of the studies varied. Various reference datasets for weight related outcomes were used, which led to different definitions of obesity in children. The BMI z-score was often used as an outcome measure in children, however, its role in childhood obesity has been challenged. In children aged less than 9 years, BMI z-score is a weak to moderate predictor of total fat mass and body fat percentage [38]. In addition, it is also a weak predictor of total body fat changes over time with poor specificity [39,40]. Hence, in clinical practice, changes in body composition among obese children should not solely be monitored using BMI z-score.

Our meta-analyses showed the effects of interventions on BMI z-score, waist circumference and body fat measurements were inconclusive for the management of childhood obesity. We are not able to make comparison with a previous review [9] as they pooled standard BMI data rather than BMI z-score, waist circumference or body fat. Further their review also included adolescents. The inconclusive findings from our meta-analyses could be due to differences in intervention strategies.

Our review provides insight into the impact of lifestyle interventions combined with behavioural strategies in reducing the weight related outcomes among overweight and obese schoolchildren. The involvement of family members in the treatment of overweight and obesity could not be overemphasised. The intervention one chooses would depend on the resources in the school and the community. Using dedicated personnel to deliver the intervention was effective, but the cost and human resources demand would be high. In addition, strategies attempting to reduce unhealthy behaviours such as reducing sedentary behaviours and adopting healthy dietary intake seem to be more effective. Parents could be trained and empowered to promote the lifestyle changes required for the management of obesity in children [41].

This review included both randomized and non-randomized controlled trials to provide more comprehensive views of various interventions aimed to reduce weight related outcomes. We also evaluated the effects of intervention on waist circumference and body fat percentage not just on BMI measures as these parameters are commonly monitored in clinical practice. Several limitations need to be mentioned in this review. Even though we have employed an extensive search strategy, we limited the publications to English language only, due to limited resources. Hence, the effectiveness of the interventions could be overrepresented. Our review only included RCTs and quasi-experimental studies, and not cohort studies. The latter study design would provide a better reflection of clinical practice. Our search strategy was specific for diet, nutrition and physical activity interventions. Hence, publications that used term such as weight management were not captured. Most studies included in this review had high or unclear risk of bias with regards to the allocation concealment and blinding of the assessors. Therefore, the findings reported should be interpreted with caution. Most studies also had high or unclear risk of bias for blinding of participants and personnel. However, these were unavoidable in view of the nature of the intervention. In addition, unpublished studies were not identified, thus, publication bias is possible. The funnel plot showed presence of possible publication bias which could be attributed to studies with small sample size and possibly with negative results were not published. Cost-effectiveness of interventions were not included in this review, which is an important aspect to consider when choosing an intervention. Hence, it should be considered in future reviews.

## Conclusion

Our meta-analyses showed that current interventions for the management of obesity among schoolchildren on weight related outcomes were inconclusive. However, based on the narrative synthesis, the role for behavioural lifestyle interventions with interdisciplinary team approaches and family involvement is crucial to curb obesity among schoolchildren. But more robust studies are needed to determine its effectiveness.

## Supporting information

S1 File PRISMA checklist. (DOC)
